# 12-Deoxyphorbol Esters Induce Growth Arrest and Apoptosis in Human Lung Cancer A549 Cells Via Activation of PKC-δ/PKD/ERK Signaling Pathway

**DOI:** 10.3390/ijms21207579

**Published:** 2020-10-14

**Authors:** Ju-Ying Tsai, Dóra Rédei, Judit Hohmann, Chin-Chung Wu

**Affiliations:** 1 Graduate Institute of Natural Products, Drug Development and Value Creation Research Center, Kaohsiung Medical University, Kaohsiung 80708, Taiwan; r040114@gap.kmu.edu.tw; 2 Department of Pharmacognosy, Interdisciplinary Excellence Centre, University of Szeged, Eötvös u. 6, H-6720 Szeged, Hungary; redei@pharmacognosy.hu (D.R.); hohmann@pharm.u-szeged.hu (J.H.); 3 Department of Medical Research, Kaohsiung Medical University Hospital, Kaohsiung 80708, Taiwan

**Keywords:** prostratin, phorbol ester, PKC-δ, PKD, ERK

## Abstract

Prostratin, a non-tumor promoting 12-deoxyphorbol ester, has been reported as a protein kinase C (PKC) activator and is shown to have anti-proliferative activity in certain cancer cell types. Here we show that GRC-2, a prostratin analogue isolated from *Euphorbia grandicornis*, is ten-fold more potent than prostratin for inhibiting the growth of human non-small cell lung cancer (NSCLC) A549 cells. Flow cytometry assay revealed that GRC-2 and prostratin inhibited cell cycle progression at the G2/M phase and induced apoptosis. The cytotoxic effect of GRC-2 and prostratin was accompanied by activation and nuclear translocation of PKC-δ and PKD as well as hyperactivation of extracellular signal-related kinase (ERK). Knockdown of either PKC-δ, PKD or ERK significantly protected A549 cancer cells from GRC-2- and prostratin-induced growth arrest as well as apoptosis. Taken together, our results have shown that prostratin and a more potent analogue GRC-2 reduce cell viability in NSCLC A549 cells, at least in part, through activation of the PKC-δ/PKD/ERK pathway, suggesting the potential of prostratin and GRC-2 as anticancer agents.

## 1. Introduction

Phorbol esters are naturally occurring diterpenoids isolated from plant species belonging to the *Euphorbiaceae* and *Thymelaeaceae* families [1]. Phorbol 12-myristate 13-acetate (TPA), the prototype of phorbol ester, is known as a potent tumor promoter in the mouse skin model. However, phorbol esters with different ester substituents and lipophilicities exhibit different tumor-promoting activity. It is known that high tumor- promoting activity by phorbol esters requires longer length for acyl residues located at C-13 and C-12 [2]. In contrast, 12-deoxyphorbol derivatives with short-chain substituents, such as prostratin (12-deoxyphorbol 13-acetate) and 12-deoxyphorbol 13-phenylacetate, are not tumor-promoting [3,4]. In fact, some phorbol derivatives exhibit potent anti-proliferation activity against cancer cells and are considered promising anticancer agents [2,5]. For example, prostratin reduces cell viability and proliferation in certain kinds of cancer cells, including acute myeloid leukemia, breast cancer, and pancreatic cancer [6,7]. In particular, prostratin significantly suppresses the burden of human pancreatic cancers in mouse xenograft models [7]. Moreover, ingenol 3-angelate, a phorbol ester-like compound has been approved for the topical treatment of actinic keratosis, which is a principal precursor of cutaneous squamous cell carcinoma [8].

PKC is a family of serine/threonine protein kinases which have been classified into three subfamilies including conventional (α, β, γ), novel (δ, ε, η, θ), and atypical (ζ, λ/ι) subfamilies [9] according to their co-factor requirements. The activation of conventional PKCs is dependent on diacylglycerol (DAG) and Ca^2+^, while novel PKCs are only dependent on DAG and atypical PKCs are DAG/Ca^2+^-independent. Phorbol esters act as potent and long-lasting DAG mimetics and thus can activate conventional and novel PKCs. PKCs play critical roles in regulating a variety of cellular responses, such as cell growth, differentiation, secretion, survival, and apoptosis [10]. The involvement of PKCs in carcinogenesis has long been recognized since the discovery of tumor-promoting phorbol esters. PKC-α and PKC-β have been linked to increased cancer cell proliferation, invasion, drug resistance, and genetic instability [11]. On the other hand, PKC-δ is one of the PKC isoforms that increases apoptosis [12]. Because of the importance of PKC in cancer progression, several PKC inhibitors have been developed and entered into clinical trials; however, these therapies have failed or even exacerbated diseases [13]. Additionally, recent studies have shown that cancer-associated mutations in PKC are generally loss-of-function, suggesting the tumor-suppressor role for PKC [13]. These findings led to the hypothesis that restoring, rather than inhibiting, the activity of PKC may be a new strategy to combat cancer [14].

In addition to PKC, PKD is also a DAG receptor, and has a high affinity to phorbol esters. Typically, PKD is activated in vivo via PKC-dependent phosphorylation of the activation loop at Ser744 and Ser748, and is usually accompanied by PKD autophosphorylation at Ser916 [15]. nPKCs are the major PKC isoforms responsible for PKD Ser744/748 phosphorylation [16], but PKC-α could activate PKD in certain contexts [17,18]. Similar to PKC, PKD is also involved in cell proliferation, survival, and differentiation. However, its precise roles in cancer are still controversial [19]. Also, the relationships between PKD and phorbol esters have been less thoroughly investigated.

## 2. Results

### 2.1. GRC-2 Is More Potent than Prostratin in Inhibiting the Cell Viability of A549 Cancer Cells by Inducing Cell Cycle Arrest and Apoptosis

**Figure 1:**
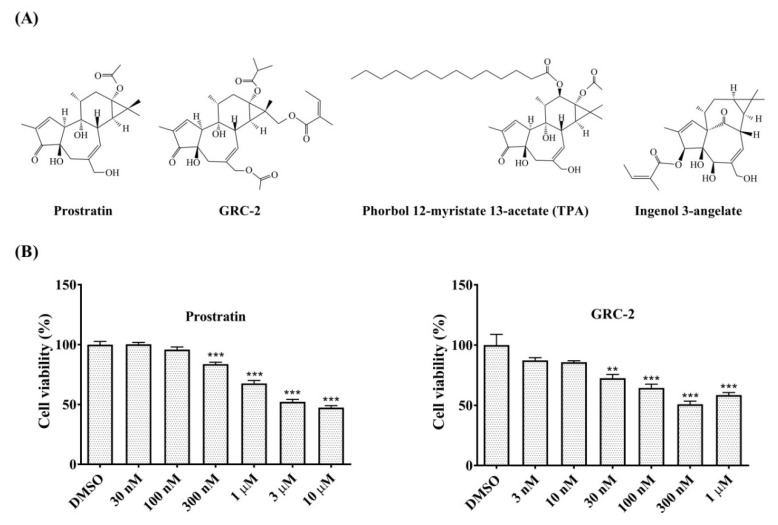
Prostratin and GRC-2 reduce cell viability of human NSCLC A549 cells. (**A**) Chemical structures of prostratin and GRC-2. (**B**) A549 cells were treated with dimethyl sulfoxide (DMSO, vehicle control), prostratin or GRC-2 for 72 h. After then, cell viability was measured by MTT assay. Results are presented as mean ± S.E.M. (*n* = 3). ** *p* < 0.01 and *** *p* < 0.001 as compared with the vehicle control.

The effects of GRC-2 and prostratin on the viability of A549 cancer cells were examined by 3-(4,5-dimethylthiazol-2-yl)-2,5-diphenyltetrazolium bromide (MTT) assay. GRC-2 decreased cell viability and reached a maximal inhibition of 49.2% at 300 nM. In contrast, prostratin caused a maximal inhibition of 47.8% at 3 μM (Figure 1B). The growth inhibitory effect of GRC-2 was also observed in three other human cancer cell lines, H1299 NSCLC cells as well as MDA-MB-231 and MCF-7 breast cancer cells, although to a lesser extent (Supplementary Figure S1). Prostratin was even weaker than GRC-2 in inhibiting growth of MDA-MB-231 cells, and had no effect on H1299 and MCF-7 cells.

**Figure 2:**
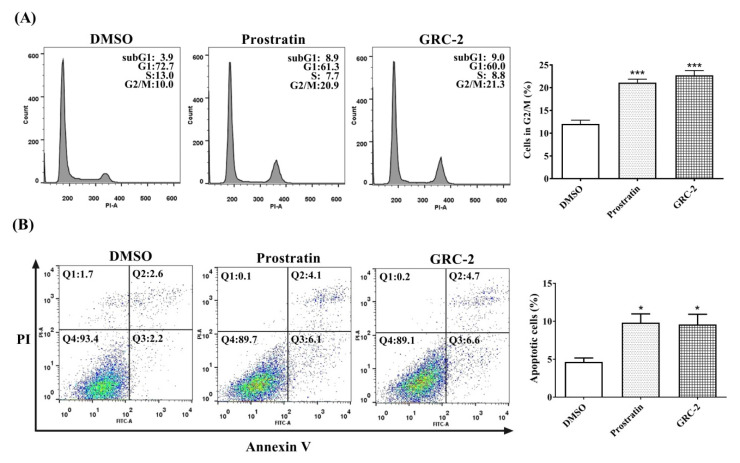
Prostratin and GRC-2 induce cell cycle arrest and apoptosis. A549 cancer cells were treated with DMSO, prostratin or GRC-2 for 72 h. (**A**) Cell cycle distribution and (**B**) apoptosis were measured by PI staining and annexin V/PI double staining, respectively. Apoptotic cells are presented in the right-lower (Q3, early apoptosis) and right-upper (Q4, late apoptosis) quadrants of the plots. Results are presented as mean ± S.E.M. (*n* = 3). * *p* < 0.05 and *** *p* < 0.001 as compared with the vehicle control.

The effects of GRC-2 and prostratin on cell cycle and apoptosis were further investigated by flow cytometry. As shown in Figure 2A, A549 cells treated with GRC-2 (300 nM) or prostratin (3 μM) for 72 h exhibited an increased G2/M population with a concomitant decrease in G0/G1 and S population, suggesting that both compounds induced cell cycle arrest at G2/M phase. In addition, the sub-G1 population showed a slight increase. Therefore, propidium iodide (PI)/Annexin V staining was performed to confirm apoptotic cell death. Figure 2B shows that both GRC-2 and prostratin increased Annexin V-positive/PI-negative cells (early apoptosis) and Annexin V-positive/PI-positive cells (late apoptosis). However, there was no significant increase in necrotic cells (Annexin V-negative/PI-positive cells) observed. Taken together, these results suggest that GRC-2 and prostratin inhibited cell viability of A549 cancer cells through induction of cell cycle arrest and apoptosis.

### 2.2. 12-Deoxyphorbol Esters Cause Activation of PKCs in A549 Cells

**Figure 3:**
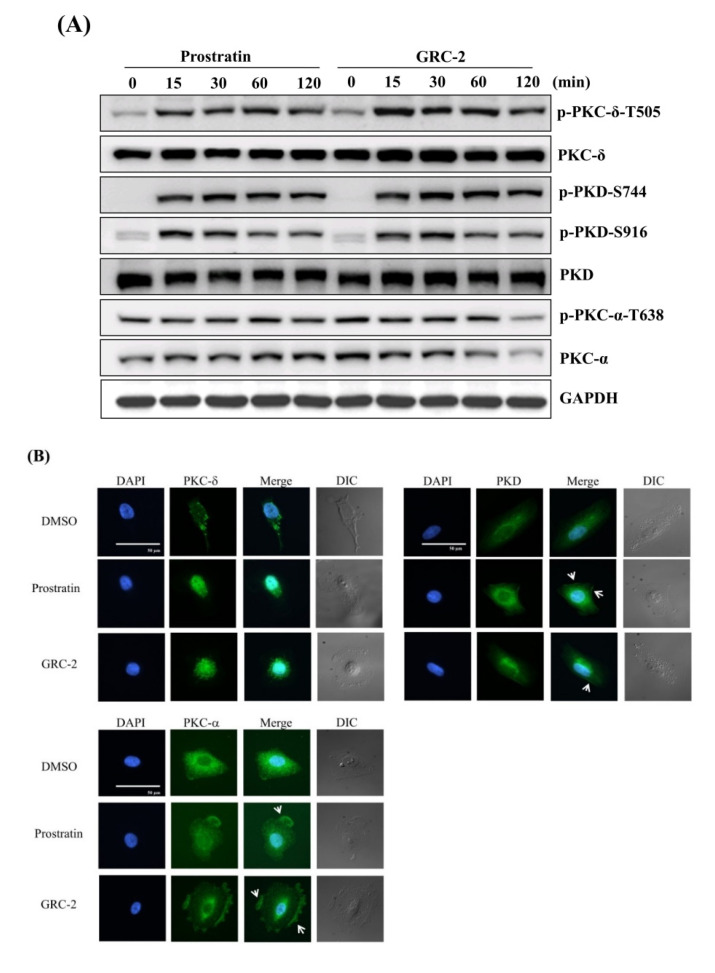
Prostratin and GRC-2 activates PKC isoforms and PKD in A549 cells. (**A**) A549 cells were treated with prostratin (3 μM) or GRC-2 (300 nM) for indicated periods. Cells were harvested and subjected to immunoblotting for PKC-δ, PKD, and PKC-α. (**B**) A549 cells were treated with prostratin (3 μM) and GRC-2 (300 nM) for 10 min. PKC-δ, PKD, and PKC-α translocation (green) were detected by immunofluorescence. Nuclei were counterstained with 4′,6-diamidino-2-phenylindole (DAPI) (blue). The images were overlapped (Merge) to determine translocation. The cell morphology was visualized with differential interference contrast (DIC) optics. Scale bar, 50 μm. White arrows indicate PKCs or PKD which were translocated to the cell membrane.

In a previous study, we demonstrated that both GRC-2 and prostratin caused PKC activation in human platelets [20]. Here we further examined the PKC-activating effect of the 12-deoxyphorbol esters in lung cancer A549 cells by measuring phosphorylation and translocation of PKC isozymes. As shown in Figure 3A, GRC-2 and prostratin induced rapid phosphorylation of PKC-δ within 15 min that lasted for 120 min. The activation of PKC-δ was also evident by the enhanced phosphorylation of Ser744 and Ser916 in PKD, which is a downstream substrate of novel PKCs [16]. In contrast, the conventional PKC isozyme PKC-α exhibited constitutive phosphorylation, which was not further induced by the 12-deoxyphorbol esters. Particularly, after a 120-min treatment of GRC-2, total and phosphorylated PKC-α levels were significantly decreased.

Membrane translocation is a hallmark of PKC activation [16,21]. By using immunofluorescence microscopy, we determined the subcellular location of PKCs in A549 cancer cells. In untreated A549 cells, PKC-δ and PKD mainly existed in the cytoplasm, while PKC-α existed in both the cytoplasm and perinuclear region. Stimulation of A549 cells with GRC-2 (300 nM) or prostratin (3 μM) induced cytoplasm-to-nucleus translocation of PKC-δ or PKD within ten minutes. In contrast, cytosolic PKC-α translocated to the plasma membrane (Figure 3B).

### 2.3. Knockdown of PKC-δ/PKD Reverses 12-Deoxyphorbol Esters-Induced Cytotoxicity against A549 Cancer Cells

**Figure 4:**
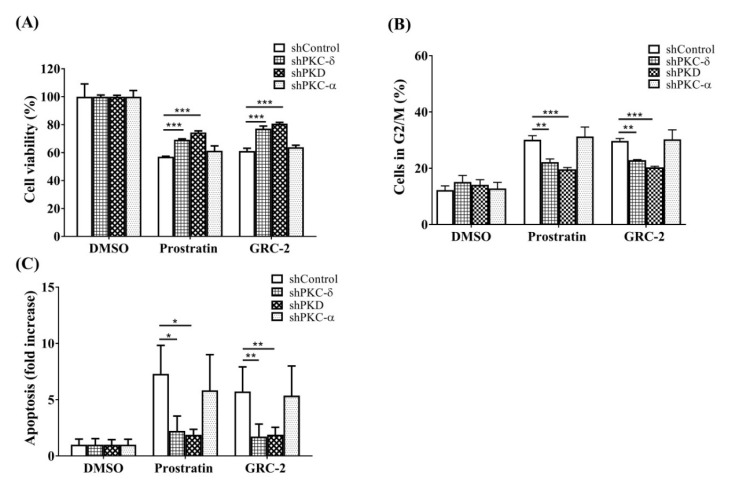

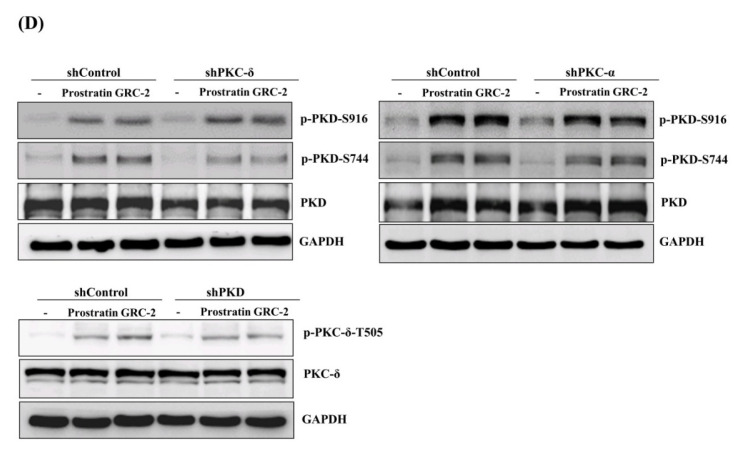
Knockdown of PKC-δ/PKD attenuates the cytotoxicity of prostratin and GRC-2 in A549 cells. A549 cells were transfected with lentiviral vectors encoding shRNA of interest or a control vector. Transfected cells were treated with DMSO, prostratin (3 μM) or GRC-2 (300 nM) for 72 h (**A**–**C**) or 15 min (**D**), then (**A**) cell viability, (**B**) cell cycle, (**C**) apoptosis, and (**D**) protein expression were measured by MTT assay, PI staining, annexin V/PI staining, and immunoblotting, respectively. The data from (**C**) are presented as fold increases in late apoptosis compared to the control vector. Results are presented as mean ± S.E.M. (*n* = 3). * *p* < 0.05, ** *p* < 0.01 and *** *p* < 0.001 as compared with the respective control.

To examine the role of PKC isoforms and PKD in the anticancer activity of the 12-deoxyphorbol esters, PKC-α, PKC-δ, and PKD in A549 cells were knocked down by specific shRNAs before treatment with GRC-2 or prostratin. The knockdown efficiency was confirmed by Western blot analysis (Supplementary Figure S2). In MTT assay, knockdown of PKC-δ and PKD reduced GRC-2-induced cytotoxicity by 41.0% and 50.5%, respectively. Similar but less pronounced effects of PKC-δ and PKD knockdown on prostratin-induced cytotoxicity were also observed. In contrast to PKC-δ, knockdown of PKC-α did not affect the cytotoxicity of both GRC-2 and prostratin. In cell cycle and apoptosis assays, knockdown of PKC-δ and PKD, but not PKC-α, partially reversed cell cycle arrest and apoptosis caused by GRC-2 or prostratin (Figure 4A–C). This result was consistent with that obtained by using pharmacological inhibitors (Supplementary Figure S3); GRC-2- and prostratin-inhibited cell viability were not affected by the PKC-α/β inhibitor Go6975, but largely rescued by the pan-PKC inhibitor GF109203.

Because PKD knockdown cells were slightly more resistant to the 12-deoxyphorbol esters than PKC-δ knockdown cells (Figure 4D), we would like to examine the effect of PKC-δ knockdown on 12-deoxyphorbol ester–induced PKD activation. As shown in Figure 4D, in PKC-δ knockdown cells, GRC-2 and prostratin was still able to induce autophosphorylation of Ser916 in PKD, even though Ser744 phosphorylation was less evident compared with that in the control cells, suggesting that GRC-2 and prostratin can activate PKD by PKC-δ-dependent and -independent manner. On the other hand, PKC-α knockdown did not significantly affect PKD phosphorylation at both sites.

### 2.4. The ERK Signaling Pathway is the Major Mediator of PKC-δ/D-Dependent Cytotoxicity of the 12-Deoxyphorbol Esters

**Figure 5:**
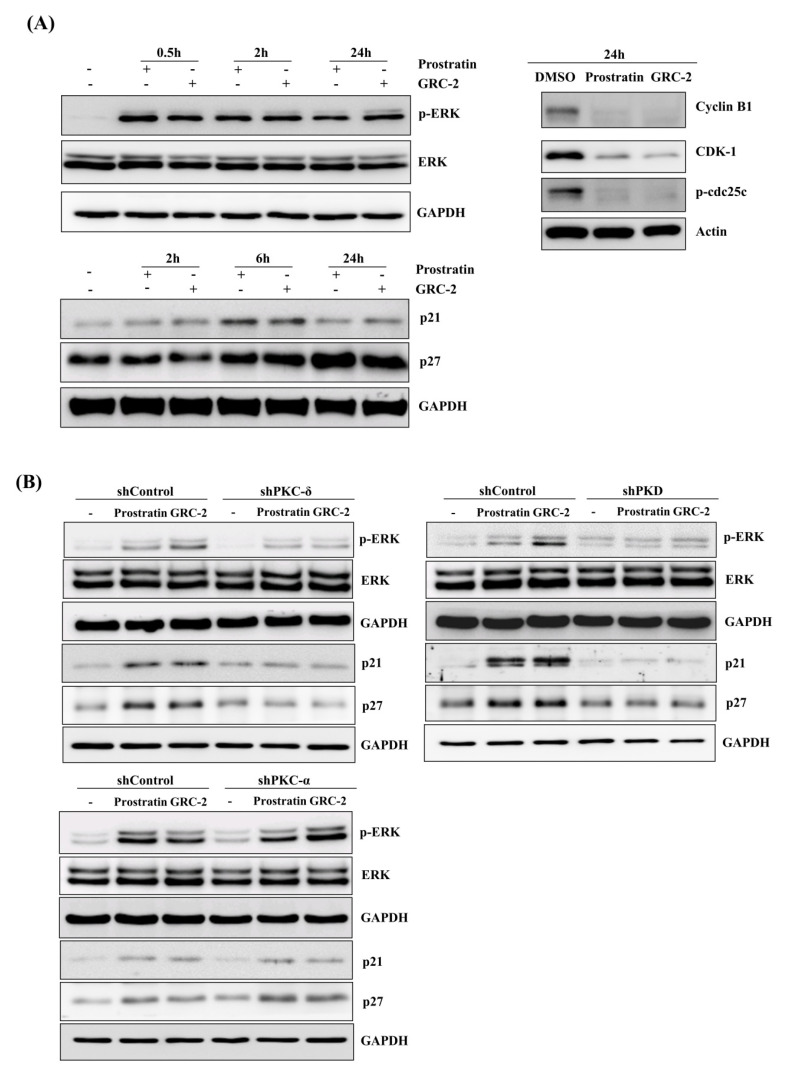
Prostratin and GRC-2 induce PKC-δ/PKD-dependent ERK activation. (**A**) A549 cells were treated with DMSO, prostratin (3 μM), or GRC-2 (300 nM) for indicated periods. Minus (-) indicates no treatment and plus (+) indicates treatment with prostratin or GRC-2. Cells were harvested and subjected to immunoblotting for ERK, p21, p27, cyclin B1, CDK1, and *p*-cdc25c. (**B**) PKC-δ-, PKD-, or PKC-α knockdown cells were treated with DMSO, prostratin, or GRC-2 for 0.5 h (ERK) and 6 h (p21 and p27), respectively, followed by immunoblotting.

Mitogen-activated protein (MAP) kinase ERK is a major downstream effector of both PKC and PKD [7,22]. Transit activation of ERK by growth factor stimuli is a prerequisite for cell proliferation; however, prolonged activation of ERK can result in cell cycle arrest, which is mediated by induction of the cyclin-dependent kinase inhibitor p21Cip/WAF1 [23,24,25]. As shown in Figure 5A, A549 cancer cells treated with GRC-2 or prostratin resulted in rapid phosphorylation of ERK, which lasted at least for 24 h. Additionally, p21 and another cyclin-dependent kinase inhibitor p27 were also induced. Upregulation of the cyclin-dependent kinase inhibitors was accompanied by decreases in cyclin B1, cyclin-dependent kinase-1 (CDK1), and phospho-cdc25c, which are related to G2-to-M transition [26].

Knockdown of PKD or, to a lesser extent, PKC-δ inhibited the 12-deoxyphorbol esters-induced ERK phosphorylation as well as p21/p27 expression (Figure 5B). In contrast, PKC-α knockdown had no significant effect.

**Figure 6:**
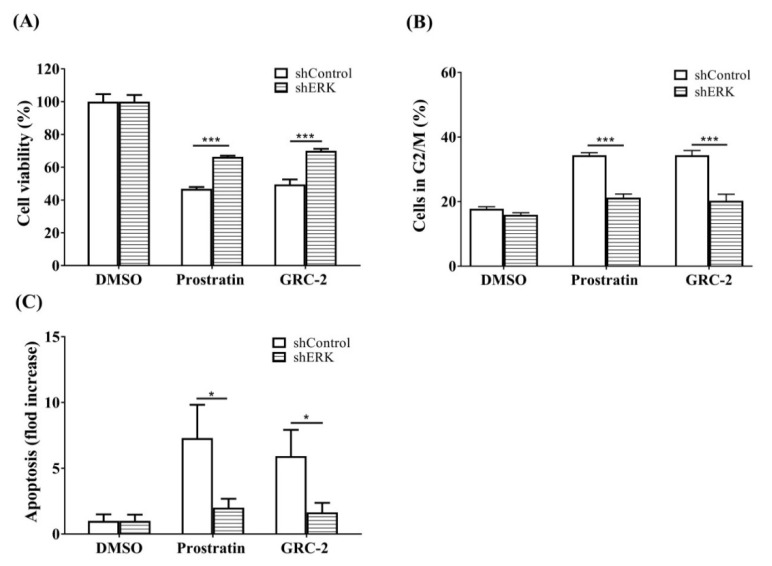
Knockdown of ERK attenuates the cytotoxicity of prostratin and GRC-2 in A549 cells. The ERK knockdown A549 cells were treated with DMSO, prostratin (3 μM) or GRC-2 (300 nM) for 72 h. (**A**) The cell viability, (**B**) cell cycle, and (**C**) apoptosis were measured by MTT assay, PI staining and annexin V/PI staining, respectively. The data from (**C**) are presented as fold increases in late apoptosis compared to the control vector. Results are presented as mean ± S.E.M. (*n* = 3) * *p* < 0.05 and *** *p* < 0.001 as compared with the respective control.

Next, ERK in A549 cancer cells was knocked down to confirm its role in mediating the anticancer activity of the 12-deoxyphorbol esters (Figure 6). The MTT assay revealed that A549 cells transfected with ERK shRNA were more resistant to both GRC-2- and prostratin-induced cytotoxicity than those transfected with the control vector. Likewise, cell cycle arrest and apoptosis caused by the 12-deoxyphorbol esters were also attenuated in ERK knockdown cells.

## 3. Discussion

Prostratin, a 12-deoxyphorbol ester, has been extensively studied for its potential use in human immunodeficiency virus (HIV) adjuvant therapy, because it is able to reduce HIV latency by activating PKC [27,28]. Recently, prostratin is also emerging as a promising anticancer agent. In acute myeloid leukemia cells, prostratin inhibited cell growth and induced differentiation by PKC-dependent ERK activation [7]. Additionally, prostratin inhibited proliferation of K-Ras-driven human pancreatic cancer cells, and suppressed tumor initiation and tumor size in a xenografted pancreatic tumor model [7]. However, the specific PKC isoforms required for the anti-tumor activity of prostratin remain to be defined. In the present study, we show that prostratin and a more potent 12-deoxyphorbol ester derivative, GRC-2, inhibit cell growth and cause apoptosis in human NSCLC A549 cells mainly through activation of PKC-δ and another DAG/phorbol ester receptor PKD. Several lines of evidence support this suggestion. First, both 12-deoxyphorbol esters elicit PKC-δ/PKD phosphorylation and nuclear translocation. In particular, it is known that nuclear translocation of PKC-δ in response to apoptotic agents is necessary and sufficient for pro-apoptotic signaling [29,30]. Second, the ability of both 12-deoxyphorbol esters to activate PKC-δ/PKD is correlated with their cytotoxic potency against NSCLC cells. Third, knockdown of either PKC-δ or PKD, but not PKC-α, significantly reduces the 12-deoxyphorbol ester-induced cell cycle arrest and apoptosis.

Previous studies have shown that hyperactivation of ERK by PKC is required for PKC activators-induced cell growth arrest and senescence [7,22], but the role of PKD is unclear. Our results indicate that in NSCLC A549 cells, the 12-deoxyphorbol esters induced marked and sustained ERK activation that is dependent on both PKC-δ and PKD. Moreover, knockdown of ERK attenuated the cytotoxicity of prostratin and GRC-2; the result is similar to that observed in PKC-δ- or PKD- knocked down cells, suggesting that hyperactivation of ERK by PKC-δ/PKD is the major mediator of the 12-deoxyphorbol esters-induced anticancer effect. Recently, Unni et al. have reported that cancers driven by mutant K-RAS will have a vulnerability to hyperactivated ERK-induced cytotoxicity [31]. This is consistent with the result here that A549 and MDA-MB-231 cancer cells harboring K-RAS mutations are more susceptible to the 12-deoxyphorbol esters than H1299 and MCF-7 cancer cells expressing wild-type K-RAS.

Although PKC activators are potentially useful for the treatment of cancer, the main challenge with systemic administration of them in patients is that various physiological responses could be elicited due to the ubiquitous expression of PKCs. One of the deleterious side effects is platelet hyperactivation. Our previous study has shown that TPA, ingenol 3-angelate, and prostratin potently induce PKC-dependent platelet aggregation with EC_50_ values of 9.5 nM, 72.5 nM, and 0.91 μM, respectively. In contrast, GRC-2 is less potent than prostratin to cause platelet aggregation (EC_50_ = 6.08 μM). In addition, the ability of GRC-2 to activate PKCs in platelets is also slightly weaker than that of prostratin. This result is contradictory to the finding in A549 cancer cells where GRC-2 is more potent than prostratin in activating PKCs. The reason for the discrepancy is unclear, but may be due to differences in the cellular context. Nevertheless, GRC-2 may benefit from a wider window for anticancer effect over platelet-stimulating effect compared with prostratin.

In conclusion, prostratin and a more potent analogue GRC-2 induce cell growth arrest as well as apoptosis in NSCLC A549 cells, and this effect is dependent on activation of the PKC-δ/PKD/ERK pathway. Our results suggest the potential use of prostratin and GRC-2 in treatment of NSCLC, in particular those that harbor KRAS mutation.

## 4. Materials and Methods

### 4.1. Reagents

GRC-2 (16-Angeloyloxy-20-acetoxy-13α-isobutanoyloxy-4β,9α-dihydroxytiglia-1,6-dien-3-one) was extracted from the aerial parts of *Euphorbia grandicornis* according the method described previously [20]. The purity of GRC-2 is above 95% according to its NMR spectrum and HPLC analysis. Prostratin was purchased from Santa Cruz Biotechnology (Santa Cruz, CA, USA). Antibodies for *p*-PKC-δ-T505, *p*-PKD-S744, *p*-PKD-S916, PKD, *p*-PKC-α-T638, *p*-cdc25c and GAPDH were purchased from Cell Signaling Technology (Beverly, MA, USA). Antibodies for PKC-δ, PKC-α, *p*-ERK, ERK, p21, p27, cyclin B1 and actin were purchased from Santa Cruz Biotechnology (Santa Cruz, CA, USA). Antibodies for CDK-1 were purchased from BD Biosciences (San Diego, CA, USA). Secondary antibodies were from Jackson ImmunoResearch Laboratories Inc. (West Grove, PA, USA).

### 4.2. Cell Lines

A549, H1299, MCF-7, and MDA-MB-231 cells were purchased from Bioresource Collection and Research Center (Hsinchu, Taiwan). Cells were cultured and maintained in DMEM-F12 medium supplemented with 10% fetal bovine serum (Hyclone, Thermo Scientific, Waltham, MA, USA), 100 U/mL penicillin and 100 μg/mL streptomycin and 1 mM sodium pyruvate (Invitrogen, Carlsbad, CA, USA). All cell cultures were maintained at 37 °C and 5% CO_2_ in a cell culture incubator.

### 4.3. Cell Viability Assay

Cells were seeded in 96 multi-well plates (TPP, Trasadingen, Switzerland) and incubated with GRC-2 and prostratin for 72 h at 37 °C and 5% CO_2_ in a cell culture incubator. MTT (3-(4,5-dimethylthiazol-2-yl)-2,5-diphenyltetrazolium bromide) was added into each well and incubated for 3 h at 37 °C and 5% CO_2_ in a cell culture incubator. Subsequently, a blue formazan crystal was dissolved by Dimethyl sulfoxide (DMSO) and cell viability was determined spectrophotometrically at a wavelength of 550 nm.

### 4.4. Transfection

The PKC-δ, PKD, PKC-α, ERK and p38 shRNA clones were purchased from the National RNAi Core Facility (Academia Sinica, Taipei, Taiwan) and were used to transfect A549 cells. A549 cells were seeded at 2.5 × 10^5^ cells per well of a 6 well plate and incubated until they reached 60–80% confluence. According to manufacturer’s protocol, 1 μg of plasmid DNA and 3 μL Lipofectamine 2000 (Invitrogen, Carlsbad, CA, USA) were mixed in 200 μL Opti-MEM I Medium (Hyclone, Thermo Scientific, Waltham, MA, USA) and incubated at room temperature for 20 min. Then, the mixture was incubated with cells for 24 h at 37 °C and 5% CO_2_ in a cell culture incubator. After 24 h, the medium was replaced with fresh medium supplemented with 10% fetal bovine serum. To obtain stable clones, cells were incubated in growth medium containing 2 μg/mL puromycin for 14 days.

### 4.5. Western Blotting

Cells were seeded in a 6 cm dish (TPP, Trasadingen, Switzerland) and incubated with GRC-2 and prostratin at 37 °C and 5% CO_2_ in a cell culture incubator. Cell extracts were harvested in lysis buffer (50 mM Tris, 150 mM NaCl, 1 mM EDTA, 1 mM EGTA, 1% Triton-X100, 1 X protease inhibitor and 1 X phosphatase inhibitor) and the protein concentration was determined with protein assay dye reagent (Bio-Rad Laboratories, Richmond, CA, USA). Protein extracts were separated by SDS-PAGE and transferred to a nitrocellulose membrane. The membrane was blocked with 5% BSA for 1 h and incubated with specific primary antibody for overnight. Then, the membrane was incubated with HRP-conjugated secondary antibody for 1 h. Finally, the membrane was probed by the chemiluminescence detection kit (Millipore, MA, USA) and visualized with a LAS-4000 mini imaging system (Fujifilm, Tokyo, Japan).

### 4.6. Immunofluorescence Staining

Cells were seeded in the glass coverslips (corning, New York, NY, USA) and incubated with GRC-2 and prostratin for 10 min at 37 °C and 5% CO_2_ in a cell culture incubator. Cells were fixed with 4% Paraformaldehyde buffer for 20 min and permeated by 0.05% Triton X-100. After washing, the cells were incubated with blocking buffer (37589, Thermo Scientific, Waltham, MA, USA) for 1 h. Subsequently, the cells were stained with the 1st antibody overnight and washed by ice-cold 1 X PBS. The cells were stained with the 2nd antibody and Hoechst 33258 (1 μg/mL) for 1 h. After washing, the cells were fixed on a microscope slide with gelatin (Sigma, St. Louis, MO, USA). Finally, the cells were examined by Cell^R^ fluorescence microscope system with Xcellence software (Olympus, Tokyo, Japan).

### 4.7. Apoptosis Assay

Apoptotic cells were detected by Dead Cell Apoptosis kit with Alexa^®^ Fluor 488 annexin V and PI (MP13241, Invitrogen, Carlsbad, CA, USA). According to manufacturer’s protocol, cells were seeded in 6 cm dish (TPP, Trasadingen, Switzerland) and incubated with GRC-2 and prostratin for 72 h at 37 °C and 5% CO_2_ in a cell culture incubator. The cells were harvested and resuspended in annexin V binding buffer. The cells were incubated with Annexin V-FITC and PI for 15 min. Subsequently, the cells were measured and recorded by a FACScan flow cytometer (BD Biosciences, Bedford, MA, USA).

### 4.8. Cell Cycles Assay

Cells were treated with GRC-2 and prostratin for 72 h at 37 °C and 5% CO_2_ in a cell culture incubator. The cells were fixed in cold 70% alcohol at 4 °C overnight; then, the fixed cells were stained in a propidium iodide solution (20 μg/μL, sigma, St. Louis, MO, USA) which contained 0.1% Triton X-100 and 0.2 mg/mL RNase for 15 min in a dark room at 37 °C. Cell cycle analysis was detected by FACScan flow cytometer (BD Biosciences, Bedford, MA, USA).

### 4.9. Statistical Analysis

Results are expressed as the mean ± standard error of the mean (SEM). Statistical significance was calculated by one-way analysis of variance (ANOVA); *p* < 0.05 was considered statistically significant.

## Supplementary Materials

Supplementary materials can be found at https://www.mdpi.com/1422-0067/21/20/7579/s1.

## Abbreviations

PKC	Protein kinase C
NSCLC	Human non-small cell lung cancer
PKD	Protein kinase D
ERK	Extracellular signal-related kinase
TPA	Phorbol 12-myristate 13-acetate
DAG	Diacylglycerol
MTT	3-(4,5-Dimethylthiazol-2-yl)-2,5-diphenyltetrazolium bromide
PI	Propidium iodide
DAPI	4′,6-diamidino-2-phenylindole
DMSO	Dimethyl sulfoxide
MAP	Mitogen-activated protein
CDK1	Cyclin-dependent kinase-1
HIV	Human immunodeficiency virus
