# Supplementary material for: 12-Deoxyphorbol Esters Induce Growth Arrest and Apoptosis in Human Lung Cancer A549 Cells Via Activation of PKC-δ/PKD/ERK Signaling Pathway
Source: Int J Mol Sci. 2020 Oct 14;21(20):7579. doi: 10.3390/ijms21207579 (PMC7589005; doi:10.3390/ijms21207579)
Supplement: Supplementary file 1 [file ijms-21-07579-s001.pdf]

Supplementary data

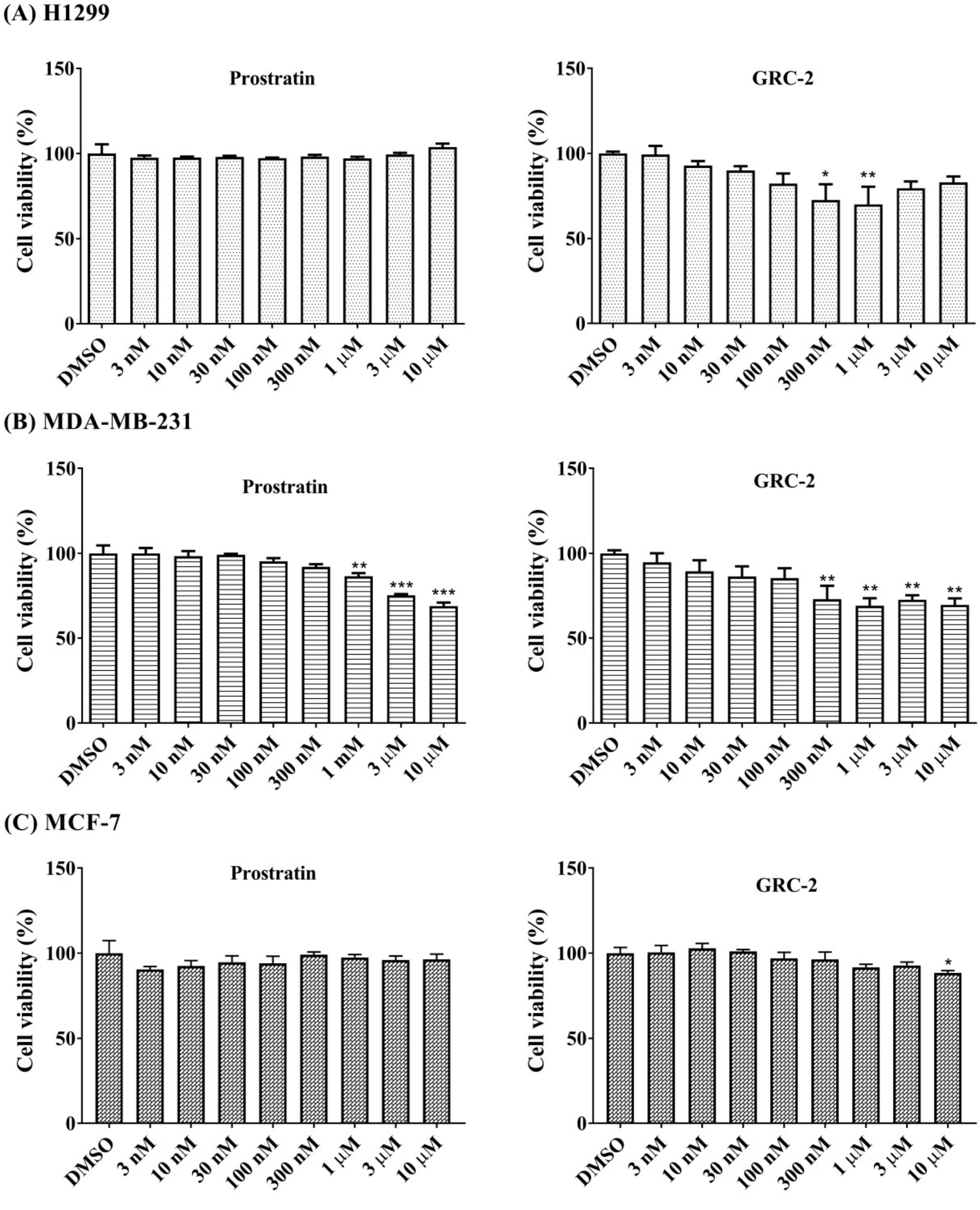

Figure S1. Effect of Prostratin and GRC-2 on the cell viability of different types of cancer cells. H1299, MDA-MB-231, and MCF-7 cells were treated with DMSO, prostratin, or GRC-2 for 72 h. Cell viability was determined by MTT assay. Results are presented as mean ± S.E.M. (n = 3). *$P < 0.05$ and **$P < 0.01$ as compared with the vehicle control.

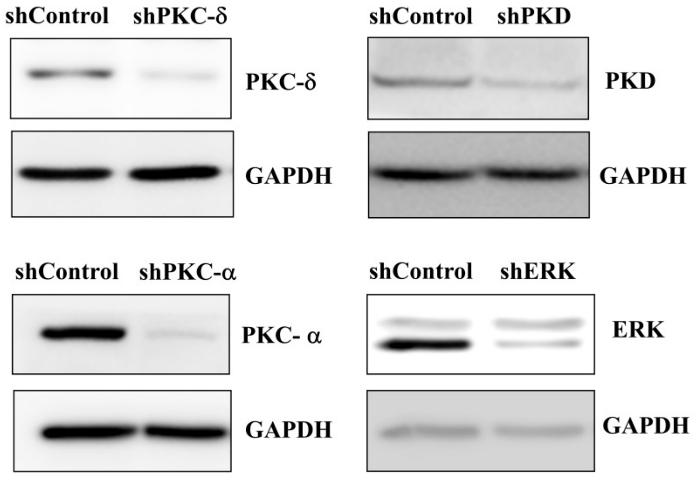

Figure S2. PKCs, PKD, and ERK expression are effectively reduced by using RNA interference. A549 cells were transfected with lentiviral vectors encoding shRNA of interest or a control vector. The protein expression of PKC-δ, PKD, PKC-α, and ERK were detected by immunoblotting.

(A)

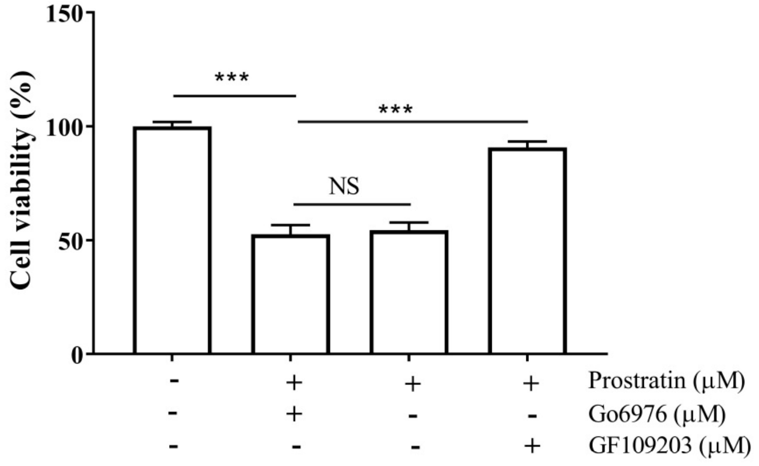

(B)

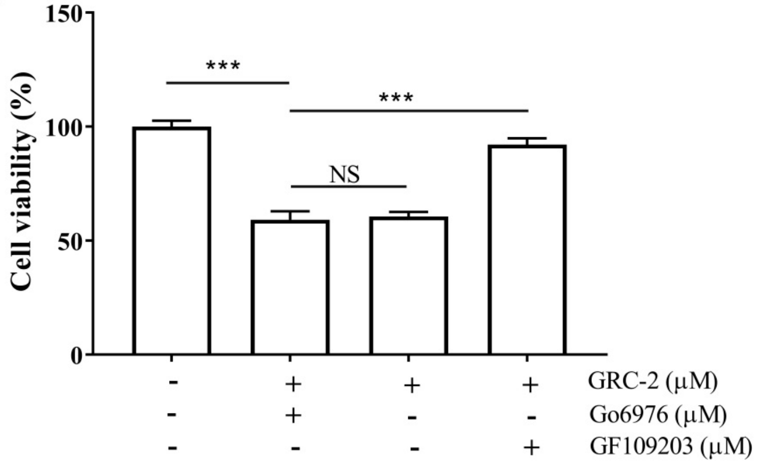

Figure S3. Effects of pharmacological inhibitors of PKC on the cytotoxicity caused by prostratin and GRC-2.

A549 cells were pretreated with Go6976 (0.1 μM) or GF109203 (1 μM) for 1 h, respectively. After then, cells were incubated with prostratin (3 μM) and GRC-2 (300 nM) for 72 h. The cell viability was measured by MTT assay. Results are presented as mean ± S.E.M. (n = 3). ***P < 0.001 as compared with the indicated groups.
